# Neonatal jaundice assessment across all skin tones: finding the gaps between guidelines and practice

**DOI:** 10.1136/archdischild-2025-330053

**Published:** 2026-04-01

**Authors:** Jacques Cornwell, Mailis Michaud Maturana, Joanna Mena, Kamla Pillay, Judith Meek, Cheentan Singh, Sasha R Howard, Eva Loucaides, The London REACH Network Collaborative, Yomna Ahmed

**Affiliations:** 1 The London Research, Evaluation and Audit for Child Health (REACH) Network, London, UK; 2 University College London Hospitals NHS Foundation Trust Neonatal Care, London, England, UK; 3 North Middlesex University Hospital Paediatrics, London, England, UK; 4 Queen Mary University of London William Harvey Research Institute, London, England, UK; 5 Queen Mary University of London, London, England, UK

**Keywords:** Jaundice, Neonatology, Paediatrics, Healthcare Disparities, Child Health Services

 The UK National Institute for Health and Care Excellence (NICE) guideline ‘Jaundice in newborn babies under 28 days’ (CG98) provides an evidence-based framework for this common condition.^1^ However, the 2023 National Health Service (NHS) Race and Health Observatory report highlighted how current neonatal jaundice practice contributes to race-related healthcare disparities.^2^ Infants from black, South Asian or other non-white ethnic groups are at a disproportionate risk of complications from severe jaundice, making up 50% of all cases of kernicterus despite representing only 25% of UK live births.^3^ Furthermore, evidence suggests that transcutaneous bilirubin (TcB) measurements are less precise in neonates with dark skin. However, these findings are derived from studies with small sample sizes and additional data are needed to robustly assess the impact of skin tone on TcB and serum bilirubin (SBR) concordance.^2^

As part of the Research, Evaluation and Audit for Child Health (REACH) Network, we analysed London-wide neonatal jaundice practices in preparation for our pan-London multicentre observational study to investigate the detection and assessment of jaundice in neonates of different skin tones: BiliNEST.^4^ Resident doctors at all London NHS sites offering postnatal care were contacted to share local clinical practice guidelines (CPGs) and complete a survey. Between January–December 2024, we received survey responses from 77% (20/26) of hospitals and obtained 19 distinct CPGs covering 88% (23/26) of hospitals.

Our analysis revealed inconsistencies between NICE guideline recommendations, local CPGs and reported clinical practice (table 1). NICE guidance, updated in 2023, acknowledges that jaundice may be harder to detect visually in darker skin, but advises against bilirubin measurements in non-visibly jaundiced babies. Nevertheless, 10% (2/20) of sites reported TcB measurements for all newborns and 30% (6/20) performed targeted screening according to risk factors (table 1). Furthermore, 9% of CPGs (2/23) suggested lower thresholds for bilirubin measurements in darker-skinned babies, for example, ‘jaundice may not easily be seen in darker skin tones—therefore, have an earlier threshold to check the bilirubin’.

**Table 1:** Evaluation of local CPG and/or survey-reported clinical practice compliance with recommendations from NICE guideline CG98^1^

NICE CG98 recommendation^1^	Local CPG compliance (n=23*)	Surveyed practice compliance (n=20)	Comments
Do not measure bilirubin levels routinely in babies who are not visibly jaundiced (1.2.7)	**83%** (**19/23**)	**60%** (**12/20**)	Significant discrepancy between CPGs and clinician-reported practice: **CPGs:** 4/23 CPGs advised routine bilirubin measurement for babies with specific risk factors: prematurity (4/4), exclusive breastfeeding (1/4) or a sibling requiring phototherapy (1/4). **Survey:** Clinicians at 2/20 sites reported routine bilirubin testing on all babies. 6/20 reported targeted bilirubin testing for babies with specific risk factors: prematurity (5/6), weight loss (3/6), cephalhaematoma (2/6), low birth weight (2/6), bruising (1/6) or a sibling with significant jaundice (1/6).
If a transcutaneous bilirubinometer measurement indicates a bilirubin level greater than 250 µmol/L, measure the SBR to check the result, (…) use SBR measurement if bilirubin levels are at or above the relevant treatment thresholds for their age (1.2.16)	**30%** (**7/23**)	**15%** (**3/20**)	Significant discrepancy between CPGs and clinician-reported practice: **CPGs:** Of the 16/23 non-compliant CPGs, 7/16 recommended SBR testing for TcB results within 20–50 µmol/L of the treatment threshold (2–5 ‘boxes below the line’), 3/16 did not specify a threshold, 4/16 solely mentioned >200 or >250 µmol/L and 2/16 gave gestation-specific thresholds. **Survey:** Of the other 17/20 respondents, most (14/17) reported SBR testing was triggered by TcB results within 20–50 µmol/L of the treatment threshold, 2/17 only for TcB readings >250 µmol/L and 1/17 at gestation-specific thresholds.
Be aware that changes to skin pigmentation because of hyperbilirubinaemia may be harder to see in darker skin (1.2.5)	**52%** (**12/23**)		Of the remaining 11/23 CPGs, 5/11 made some reference to skin tone but did not explicitly state that visually detecting jaundice may be more challenging in babies with darker skin.
Examine the sclerae and gums, and press lightly on the skin to check for signs of jaundice in ‘blanched’ skin (1.2.5)	**83%** (**19/23**)		The remaining 4/23 CPGs did not specify the recommended technique for visually assessing newborns for jaundice.
Offer parents or carers information about neonatal jaundice (…) backed up by written information (1.1.1)		**40%** (**8/20**)	12/20 survey respondents reported no parental leaflet on newborn jaundice was routinely in use.

Where compliance was assessed both on the basis of CPGs and surveyed practice, results from each are reported separately in the two middle columns. Grey cells are shown where compliance was only assessed via either the CPG or surveyed practice.

* 7/23 (30%) of CPGs had not been reviewed within the last 3 years.

CG, clinical guideline ; CPG, clinical practice guideline; NICE, National Institute for Health and Care Excellence; SBR, serum bilirubin; TcB, transcutaneous bilirubin.

Moreover, we found that clinicians are adopting precautionary practices that deviate from national standards, potentially to compensate for diagnostic uncertainty. While NICE recommends confirmatory SBR only when TcB levels exceed either 250 µmol/L or the treatment threshold, only 15% (3/20) of practitioners followed this. Instead, 75% (15/20) adopted a more cautious approach, performing SBR testing for TcB levels 20–50 µmol/L below the treatment threshold. Given the suspected variance of TcB accuracy in pigmented skin, this practice may reflect a lack of clinical confidence in non-invasive tools for diverse populations.

Most concerningly, despite the explicit warning from NICE that ‘[…] hyperbilirubinaemia may be harder to see in darker skin’, only half of CPGs (52%, 12/23) included this crucial detail (table 1).

This evaluation highlights a disconnect between the known risks of severe jaundice in black and South Asian infants and the guidance provided to frontline clinicians. Half of local guidelines fail to highlight visual assessment risks, and clinicians appear to be creating informal ‘safety margins’ regarding TcB accuracy. There is an urgent need for consensus on specific protocols for jaundice assessment in darker skin tones to close this safety gap.

## Data Availability

Data and materials related to the referenced study are available upon request from the corresponding author.
